# A population spatialization method based on the integration of feature selection and an improved random forest model

**DOI:** 10.1371/journal.pone.0321263

**Published:** 2025-04-03

**Authors:** Zhen Zhao, Hongmei Guo, Xueli Jiang, Ying Zhang, Changjiang Lu, Can Zhang, Zonghang He

**Affiliations:** 1 The Seismological Bureau of Sichuan Province, Chengdu, Sichuan, China; 2 Southwest Jiaotong University, Chengdu, Sichuan, China; National Central University, TAIWAN

## Abstract

Ascertaining the precise and accurate spatial distribution of population is essential in conducting effective urban planning, resource allocation, and emergency rescue planning. The random forest (RF) model is widely used in population spatialization studies. However, the complexity of population distribution characteristics and the limitations of the RF model in processing unbalanced datasets affect population prediction accuracy. To address these issues, a population spatialization model that integrates feature selection with an improved random forest is proposed herein. Firstly, recursive feature elimination using cross validation (RFECV), maximum information coefficient (MIC), and mean decrease accuracy (MDA) methods were utilized to select population distribution feature factors. The random forest was constructed using feature subsets that were selected via different feature selection methods, namely MIC-RF, RFECV-RF and MDA-RF. Subsequently, the feature factors corresponding to the model with the highest accuracy were selected as the optimal feature subsets and used in the model construction as input data. Additionally, considering the imbalanced in population spatial distribution, we used the K-means ++ clustering algorithm to cluster the optimal feature subset, and we used the bootstrap sampling method to extract the same amount of data from each cluster and fuse it with the training subset to build an improved random forest model. Based on this model, a spatial population distribution dataset of the Southern Sichuan Economic Zone at a 500m resolution was generated. Finally, the population dataset generated in this study was compared and validated with the WorldPop dataset. The results showed that utilizing feature selection methods improves model accuracy to varying degrees compared with RF based on all factors, and the MDA-RF had the lowest MAPE of 0.174 and the highest R^2^ of 0.913 among them. Therefore, feature factors selection using the MDA method was considered the optimal feature subset. Compared with MDA-RF, the prediction accuracy of the improved RF built on the same subset increased by 1.7%, indicating that improving the bootstrap sampling of random forest by using the K-means++ clustering algorithm can enhance model accuracy to some extent. Compared with the WorldPop dataset, the accuracy of the results predicted using the proposed method was enhanced. The MRE and RMSE of the WorldPop dataset were 57.24 and 23174.98, respectively, while the MRE and RMSE of the proposed method were 25.00 and 15776.50, respectively. This implies that the method proposed in this paper could simulate population spatial distribution more accurately.

## 1. Introduction

Population spatialization data reflect the population distribution in the objective world and fine-scale population distribution data are fundamental for urban development planning, resource allocation, public health management, disaster assessment, and emergency rescue planning [1–6]. At present, the official population figures derived from census data are usually reported at the administrative unit level (e.g., province, city, country, township) [7]. Population census data represents the total population within administrative divisions and this fails to reflect the spatial distribution and heterogeneity of the population within administrative units [8–10]. The population census data also have limitations in that they are not suitable for combination with other data (e.g., data pertaining to land use, environment, natural resource, etc.) for superposition analysis [11]. However, gridded population datasets can overcome these shortcomings because they can reflect the spatial distribution characteristics of the population [12–14]. Therefore, it is particularly important to conduct robust research on population spatialization and to allocate statistical populations to fine-grained spatial units.

Modeling factors and models are the two important aspects of building a population spatialization model. On the one hand, with the rapid development of information technology, there are more and more methods and strategies for acquiring data. Population spatialization modeling data tend to be from multiple sources and refined. There are many commonly used data, such as point-of-interest (POI), digital elevation model (DEM), mobile signaling, nighttime light data, land cover data, Wechat, Twitter, etc [15–20]. When a single class factor is used as the only variable, analysts may produce an overestimation or an underestimation, or allocate to inappropriate locations due to the default assumption that the same category has the same attractiveness [21,22]. Hence, using a combination of multiple factors has become the new trend. However, using data obtained from multiple sources for constructing a spatial population model results in dimensional issues, long computing times, and reduced prediction accuracy. Therefore, understanding how to select the feature factors with strong correlations with population distribution is essential before commencing with the modeling. In studies of population spatialization, most scholars use the person correlation coefficient method to select relevant factors related to population distribution [23]. This method can measure the linear correlation between variables, but it cannot effectively measure the nonlinear relationships between variables when there is a non-linear relationship between the variables in the dataset. The spatial distribution of population is a nonlinear and complex problem that involves multiple influencing factors. Hence, scholars often use other feature selection methods to extract demographic distribution characteristic factors. For instance, Wang *et al.* [4] used the low-variance filtering feature selection method to feature extraction, and features with variance values greater than 0.8 were selected as explanatory variables and included in the model construction. The random forest model constructed has a RMSE of 9.8. Chen *et al.* [12] combined the Pearson correlation coefficient method, variance threshold selection method, relief feature selection method, and recursive feature elimination method for feature screening, and many researchers in other fields have introduced different feature selection methods to optimize the feature factors. Lin *et al.* [24] proposed a method for screening spatial time-delayed traffic series based on the maximal information coefficient, and combined the K-nearest neighbor method with the support vector regression method to predict traffic flow. The results showed that the proposed method had better performance than the traditional method. Kemal *et al.* [25] proposed the use of the recursive feature elimination using cross validation (RFECV) and stability selection (SS) methods to screen the causes of heart disease from different datasets, and used gradient boosted machines (GBM), Naive Bayes (NB), and RF algorithms to predict heart diseases. The results revealed that the accuracy of GBM prediction using the attributes screened by the RFECV improved by 14.81% and 6.18% compared to the Single Proton Emission Computed Tomography and Statlog Heart Disease datasets. Šandera *et al.* [26] used mean decrease accuracy (MDA) to select the most suitable prediction factor for detecting changes in permanent grassland and cultivated land, which yielded a detection accuracy of > 95%. Thus, the prediction accuracy of models can be considerably improved using feature factors selected via robust feature selection methods.

On the other hand, the population prediction accuracy is limited by the algorithms. At present, the most commomly used technique is statistical modeling. Statistical modeling methods establish population spatialization models based on the weighted relationships between various auxiliary data and the spatial distribution of the population. Representative methods include multiple linear regression (MLB) [27,28], geographically weighted regression (GWR) [9,29–31], spatial autoregression [32,33], etc. Moreover, many statistical regression models are based on the assumption of there being linear relationships. However, the factors that affect the spatial distribution of population are not in a simple linear superposition relationship, and the linear assumption does not reflect the complex interrelations between population distribution and associated factors. This can lead to the inaccurate modeling of relationships. With the development of artificial intelligence, many scholars have applied machine learning methods to population spatialization studies, such as neural networks [8,34,35], extreme gradient boosting (XGBoost) [5,16,36], light gradient boosting machine (LightGBM), support vector machines (SVM) [5,16], random forest (RF) [37–40], and so on. Among them, random forest is the most widely used. Random forest integrates predictions from multiple decision trees, thus avoiding overfitting [41]. In addition, random forest adopts two random processes (sample randomness and feature randomness), thereby improving the model’s robustness and generalization ability [42]. However, the spatial distribution of population is not an equilibrium, i.e., there are the small proportions of sparse and densely populated areas. Due to the randomness of sampling, randomly sampled data may predominantly come from the majority category in certain probability scenarios, leading to a non-representative sample distribution, resulting in a lower prediction accuracy of the model. To address this issue, some scholars have proposed optimizing random forest models during data preprocessing. The main approach is to adjust the data distribution in the dataset to reduce data imbalances. This can be achieved through resampling or data grouping techniques [43]. Resampling is usually divided into oversampling and under-sampling. Oversampling, which involves adding minority class samples to the dataset, with typical algorithms including synthetic minority oversampling technique (SMOTE) [44], Borderline-SMOTE [45], and adaptive synthetic sampling (ADASYN) [46]. Under-sampling involves removing majority class samples, with common algorithms including random under-sampling, Tomek Links, and clustering based under-sampling [47]. Data grouping refers to classifying imbalanced datasets according to certain rules of classification and extracting samples from each class for fusion based on certain sampling principles.

In response to this issue, this paper proposes a population spatialization model that combines feature selection and an optimized random forest algorithm. Firstly, MIC, RFECV, and MDA methods were used for extracting feature factors. Then, the feature subsets were selected, using different feature selection methods to construct different random forest models, and their prediction accuracies were compared to obtain the optimal feature subset. Subsequently, considering the imbalanced distribution of population spatial distribution, the K-mean++ clustering algorithm was used to improve bootstrap sampling and build an improved random forest model. Finally, the population spatialization model was constructed based on the optimal feature subset and improved random forest, and a spatial population distribution dataset of the Southern Sichuan Economic Zone at 500 m resolution was generated. At the same time, the dataset developed in this study was compared with the WorldPop dataset [48,15] for verification of its accuracy.

## 2. Methodology

**Fig 1:**
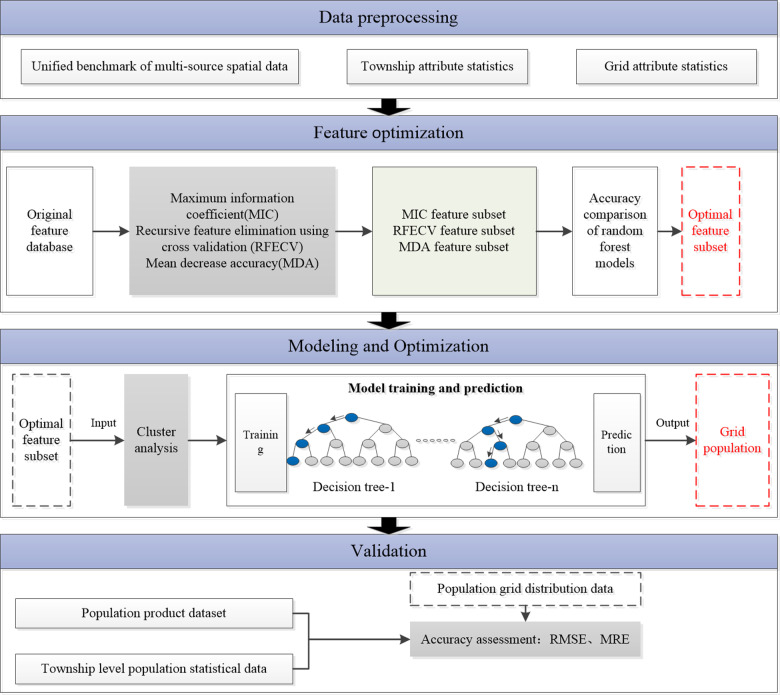
Flowchart of population spatialization.

This study is divided into four parts, including data processing, feature optimization, modeling and optimization, and validation, as shown in Fig 1. Firstly, during the data processing phase, all data were unified into the same coordinate system, and attribute statistics were conducted based on township and grids, including average elevation, average slope, average fluctuation, average aspect, average lighting brightness, percentage of different land cover types, and average kernel density of various POIs. During the feature optimization phase, various feature selection methods -MIC, RFECV, and MDA- were applied to conduct feature factor screening, and different random forest models were constructed using the selected feature subsets. Subsequently, the feature factor corresponding to the model with the highest accuracy was selected as the optimal feature subset. In the modeling and optimization stage, the K-Means++ clustering algorithm was utilized to cluster the optimal feature subset. Random forest was chosen as the modeling method. Simultaneously, the bootstrap sampling method was used to randomly extract an equal number of subsamples from each class, and the subsamples were fused as training samples to address the imbalance of samples, for the purpose of building an optimal model. Furthermore, using an optimized random forest model based on the optimal feature subset, we predicted and modeled the spatial distribution of the population in the Southern Sichuan Economic Zone for the year 2020. We obtained the population spatialization dataset at 500 m resolution. Finally, the obtained population grid dataset was compared with the WorldPop dataset at the township level for comparative analysis and accuracy verification.

### 2.1. Feature selection

The main purposes of feature selection are to select the features (i.e., factors) that have an important impact on response variables from the original feature set by using a feature screening algorithm, to eliminate the features that have no impacts or negative impacts, to reduce the number of useful factors, and to improve the computational speed of training and model accuracy [49]. The pearson correlation coefficient method has been used by most researchers in population spatialization studies to screen feature factors related to population distribution. This method can discern a linear correlation between variables, but it cannot measure nonlinear relationships between variables. The spatial distribution of population is a nonlinear and complex problem that involves multiple influencing factors. Considering only linear relationships for feature selection can entail the risk of losing some important nonlinear features, hence it is necessary to consider both linear and nonlinear relationships holistically. Therefore, in this article, various feature selection methods were used to identify the most suitable features, including recursive feature elimination using cross validation, maximum information coefficient, and mean decrease accuracy.

#### 2.1.1. Maximum information coefficient.

The maximum information coefficient (MIC), a filter-type feature selection method, can measure the degree of linear or nonlinear correlations between two variables based on the following principle: If a correlation exists between a factor (*X*) of population distribution and the population density response (*Y*), their variables are divided by grids. Then, the probability distribution density at the grid edge and the joint probability distribution density of each point in the grid are calculated. The mutual information value of *X* and *Y* is subsequently calculated, and its maximum value is determined. Finally, the maximum mutual information coefficient is obtained using the MIC calculation formula with normalization of the maximum mutual information [24].


$$I\left(X,Y\right)=\int P(X,Y)\log_{2}\frac{P(X,Y)}{P(X)P(Y)}\approx \sum_{X,Y}P(X,Y)\log_{2}\frac{P(X,Y)}{P(X)P(Y)}$$ (1)

$$MIC(X,Y)={\text{max}}_{a\times b<n_{s}^{0.6}}\frac{I(X,Y)}{\log_{2}(\min(a,b))}$$ (2)

where *P*(*X*) and *P*(*Y*) are the edge probability distribution densities; *P*(*X*, *Y*) is the joint probability distribution density; and *I*(*X*, *Y*) is the mutual information value of *X* and *Y*. *a* and *b* are the numbers of rows and columns in the grid, respectively.

#### 2.1.2. Recursive feature elimination using cross validation.

The RFECV method is an encapsulated feature selection method and performs feature screening in two stages: recursive feature elimination (RFE) and cross validation (CV). The RFE calculates the importance of all features in the first stage, and the optimal feature subset is automatically selected through CV in the second stage. Different numbers of features are sequentially selected based on calculated feature importance, and the average evaluation score of each feature subset is calculated. Finally, the feature subset with the least number of features and the highest score is obtained [25]. A RF model with default initial values of model parameters is constructed using the ten-fold cross-validation method as the base learner. The features of low importance were sequentially excluded via feature sequencing; this process was repeated, and the results were ranked in their order of importance.

#### 2.1.3. Mean decrease accuracy.

The MDA method based on a RF selector is an embedded feature selection method which randomly divides the dataset into training and test sets by proportion. The feature data of the training set are input into the RF to verify the model accuracy via CV. Then, the feature values corresponding to a given town are scrambled and input into the model with other feature data to verify model accuracy. If the model accuracy considerably decreases after the feature order is scrambled, it indicates that this feature is important.

### 2.2. Improved random forest

Random forest is a classification tree–based machine learning algorithm [50,51]. RF integrates predictions from multiple decision trees, with each tree learning from the data in a different way, thereby reducing the risk of overfitting and improving the overall model’s generalization ability. This leads to more accurate predictions, helps avoid overfitting, and exhibits higher tolerance to outliers and noise [52–54]. However, the random forest model has certain limitations when dealing with low-dimensional imbalanced datasets. This is because the random sampling process using bootstrap with replacement exacerbates the imbalance in the dataset. However, population spatial distribution exhibits unevenness, with a few high-density and low-density areas, and a majority of moderate-density areas. As a result, there are big differences in the population data sets, causing the training set after bootstrap sampling to lack representativeness, thus leading to a decrease in the prediction accuracy of the random forest model. Therefore, to enhance the prediction accuracy of the model, this study optimized the model during preprocessing. Before randomly sampling the samples, the original dataset was first subjected to clustering using the K-Means++ algorithm. Then, an equal number of samples were selected from each cluster and merged to form a new training dataset for model construction.

#### 2.2.1. K-means++ Clustering algorithm..

The K-means clustering algorithm is an unsupervised machine learning method that is widely used due to its simplicity, ease of implementation, and fast convergence. The central principle of K-means is to group similar objects into the same cluster and assign dissimilar objects to different clusters, aiming to minimize the distance between data points within each cluster while maximizing the distance between clusters [55]. The involves randomly selecting k initial cluster centers, calculating the Euclidean distance between each cluster object and the cluster centers, assigning them to the cluster represented by the nearest center, computing the mean of all cluster objects as the new cluster center, reassigning the data to the new cluster centers, and repeating this process iteratively until the cluster centers no longer change.

**Fig 2:**
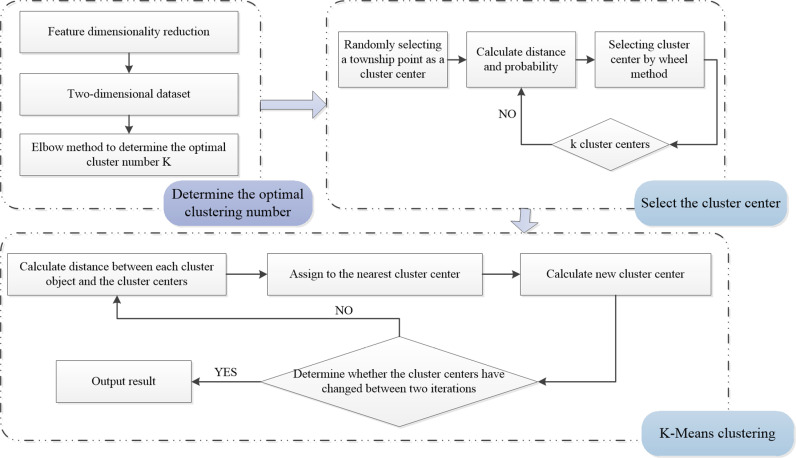
K-means++ clustering algorithm.

The K-means clustering algorithm randomly selects initial cluster centers. The result will fall into the local optimal solution if not chosen properly. Therefore, this study optimizes the selection of initial cluster centers and proposes the K-means++ algorithm (the specific process is shown in Fig 2.) Firstly, the high-dimensional feature factor data of each township in the training set are dimensionally reduced to obtain a two-dimensional dataset. The optimal number of clusters, k, is determined using the elbow method. Secondly, a random township point is selected as the first initial cluster center. Then, the distances between each township point and the cluster centers are calculated and the shortest distance is denoted as d. The probability of each township point being selected as the next cluster center is computed, and the point with the highest probability is chosen using the roulette wheel selection method as the next cluster center. This process is repeated until k cluster centers are selected.

#### 2.2.2. Random forest optimization.

**Fig 3:**
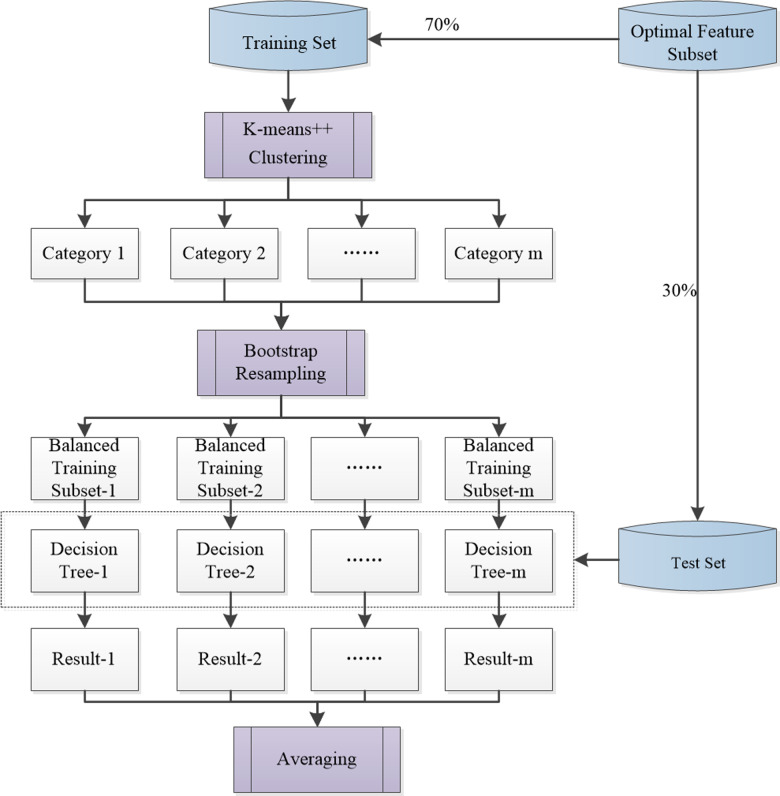
Improved random forest algorithm.

The improved random forest algorithm optimizes the model from the perspective of data preprocessing; the basic principle is shown in Fig 3. Specifically, improved random forest construction was enabled via the following steps:

First, the dataset composed of the optimal feature subset was divided into training and test sets. The selected optimal feature was considered as the clustering index. The K-means++ algorithm was used to cluster the data in the training set, and the optimal cluster number *m* was determined. The corresponding sample data were divided based on various central features.

Second, the same amount of sample data was randomly selected from various datasets to balance the original training set and reduce the impact of unbalanced datasets on model accuracy. A decision tree was then built using the new integrated training subset; multiple decision trees formed a RF.

Finally, each individual tree produces its own predicted output, and the final outputs of the whole RF model are the average of the outputs of all the trees. Therefore, the average value was used as the final result for predicting the population density.

### 2.3. Weighted random forest

The traditional random forest usually adopts the simple averaging method to deal with regression problems. However, the prediction accuracy of different base learners is different, some have higher prediction accuracy, and some have lower prediction accuracy. If these base learners are treated equally, the prediction accuracy will inevitably be affected. Therefore, in order to further improve the prediction accuracy of the random forest model, this paper proposes a weighted random forest (W-RF) based on the prediction error accuracy of the out-of-bag data of the base learners. Specific steps are as follows:

(1) The bootstrap method was used to resample the training samples, and then k training sets were randomly generated. The corresponding decision tree is further generated based on k training sets.
(2) When the i^th^ decision tree is trained, the prediction average absolute error percentage (MAPE_i_) of each decision tree is calculated using the out-of-bag data. Then the weight of the i^th^ decision tree is:

$$ACC_{i}=1\text{-}MAPE_{i}$$ (3)

(3) Calculate the weight of each CART in the random forest:

$$w_{i}=\frac{\ln\frac{ACC_{i}}{1-ACC_{i}}}{\sum_{i=1}^{n}\ln\frac{ACC_{i}}{1-ACC_{i}}}$$ (4)

(4) Calculate the predicted value of random forest model:

$$f(x)=\sum_{i=1}^{n}w_{i}T_{i}(x)$$ (5)

where $T_{i}(x)$ is the predict value of *i*th decision tree.

### 2.4. Model evaluation and accuracy validation

This study employed mean absolute percentage error (MAPE), root mean square error (RMSE) and coefficient of determination (*R*^2^) as evaluation metrics to assess the accuracy of different methods. Specifically, MAPE was used to measure the average percentage error between the predicted value and the true value. A smaller MAPE value indicates more accurate predictions by the model. RMSE is commonly used to measure the proximity between the predicted values and the actual values. A smaller RMSE value indicates higher model accuracy. R^2^ can be used to measure how well a model explains the target variable. Its values range from 0 to 1. The closer it is to 1, the better the explanation of the model. The formula of each indicator is as follows:


$$MAPE=\frac{1}{n}\sum_{i=1}^{n}\left|\frac{y_{t}(i)-y_{p}(i)}{y_{t}(i)}\right|$$ (6)

$$R^{2}=1-\frac{\sum_{i=1}^{n}{\left(y_{t}(i)-y_{p}(i)\right)}^{2}}{\sum_{i=1}^{n}{\left(y_{t}(i)-{\bar{y}}_{t}\right)}^{2}}$$ (7)

$$RMSE=\sqrt{\frac{\sum_{i=1}^{n}{\left(y_{t}(i)-y_{p}(i)\right)}^{2}}{n-1}}$$ (8)

where $y_{t}(i)$ is the actual population of the *i*th town, $y_{p}(i)$ is the predicted population, ${\bar{y}}_{t}$ is the average actual population, and *n* is the number of towns.

The proposed model integrates feature selection and improved RF for population spatialization, and it uses demographic data at township or street level. Population data from a lower level of administrative division are generally used for verifying model accuracy. However, demographics at the township or street level were the most detailed population data available here, and the accuracy of the model could not be verified at the community level (below the street level). Therefore, to validate the accuracy of the population spatialization results, this study aggregated gridded population data to townships (or streets) and calculated the errors between the estimated population and the corresponding population statistics. The results were then compared with the WorldPop dataset at the same scale and year. This study used three metrics, namely relative error (RE), mean relative error (MRE), and RMSE, to measure accuracy. The formulas for the respective metrics are as follows:


$$RE=\frac{\left|pop_{p}-pop_{t}\right|}{pop_{t}}\times 100\%$$ (9)

$$MRE=\frac{ER}{n}$$ (10)

where RE is the relative error of the township population, MRE is the mean relative error, $pop_{p}$ is the predicted value of the township population, $pop_{t}$ is the demographic data of the township, and n is the number of towns.

## 3. Study area and data

### 3.1. Study area

**Fig 4:**
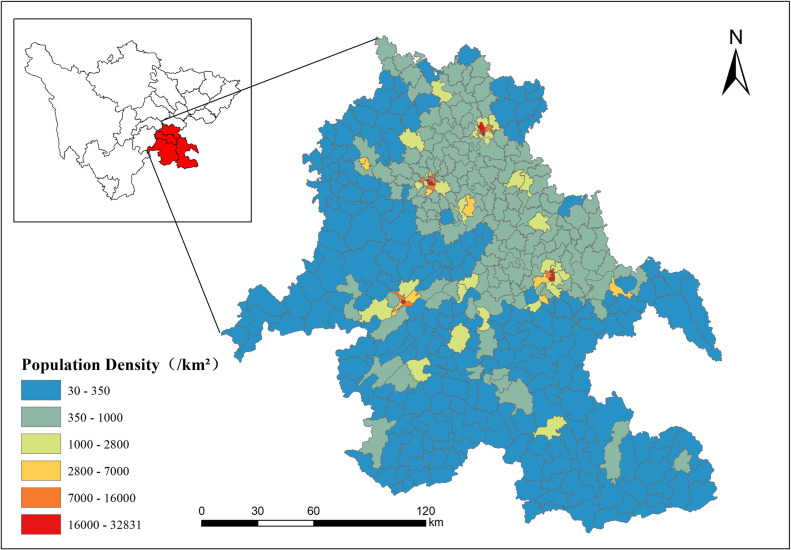
Overview of the Southern Sichuan Economic Zone (The base map is based on the administrative division data provided by the Sichuan Bureau of Surveying, Mapping and Geoinformation. ).

The Southern Sichuan Economic Zone, comprising 435 towns/streets in 28 districts and counties across the four cities of Neijiang, Zigong, Yibin, and Luzhou, was selected as the study area (Fig 4.) It is one of the five economic areas planned by the Sichuan Province in China and it is the regional economic center of the junction between Sichuan Province, Chongqing City, Yunnan Province, and Guizhou Province. The Southern Sichuan Economic Zone is a demonstration zone of modern industrial innovation and development and an important gateway to Sichuan Province to the south. It is also a demonstration zone of green development in the upper reaches of the Yangtze River. According to the statistics of the seventh national census in China, the total population of this zone was 14.472887 million, accounting for 17.3% of the total population of Sichuan Province. The total populations of Zigong City, Yibin City, Neijiang City, and Luzhou City were ~ 2.489, 4.589, 3.141, and 4.254 million, respectively. The population distribution pattern in the zone is complex, with large differences in population density—the streets have a high population density, whereas the towns have a low population density. The population density increases in areas closer to the core cities. This zone is typical of population spatialization research and was therefore selected for this study.

### 3.2. Data sources and processing

The digital elevation model (DEM), nighttime lighting data, administrative division, population census data, geographic national census, point-of-interest (POI), and WorldPop dataset were used in this study (Table 1). All spatial references of these data were unified to the Asia North Albers Equal Area Conic projection coordinate system. The acquisition and preprocessing of the data in the current study are described below.

**Table 1:** Datasets used in the study area.

Data	Source	Description
Digital elevation model data (ASTER GDEM v3	National Aeronautics and Space Administration (NASA) (https://earthdata.nasa.gov/)	The resolution is 30 m.
Nighttime lighting data (NPP-VIIRS)	National Oceanic and Atmospheric Administration (NOAA)	The resolution is 500 m.
Administrative division data	Sichuan Bureau of Surveying, Mapping and Geoinformation	The data include the boundary vector contours of cities, states, districts, counties, and towns.
Population data	The seventh national census data	The data include resident populations of cities, states, districts, counties, and towns.
Geographic national census data	Sichuan Bureau of Surveying, Mapping and Geoinformation	The data include cultivated lands, forest lands, garden lands, grasslands, buildings, roads, structures, excavated lands, deserts, and water areas; 10 first-class categories in total.
Point-of-interest data (POI)	Baidu map	The data include 14 points of interest as follows: leisure and entertainment, financial services, education and training, life services, government agencies, medical care, automobile services, companies and enterprises, transportation facilities, shopping, accommodation, tourist attractions, catering, and real estate.
WorldPop data	WorldPop official website	The resolution is 100 m.

The digital elevation model version 3 (ASTER GDEM v3) with a 30 m spatial resolution was sourced from the National Aeronautics and Space Administration (NASA). The 30 m spatial resolution DEM data were resampled to 500 m using the bilinear interpolation method, and the resampled DEM data were employed to generate the elevation, slope, aspect, and fluctuation datasets of the Southern Sichuan Economic Zone.

Administrative division data were derived from the Sichuan Bureau of Surveying, Mapping and Geoinformation, and used as a mask to extract data from within the region. The population data were sourced from the seventh national census. By spatially connecting the population data with the administrative division data of streets/towns based on their administrative names, we could calculate the population density values for each street/town.

Geographic national census data were derived from the Sichuan Bureau of Surveying, Mapping and Geoinformation. There are 10 categories included in the dataset: cultivated lands, forest lands, garden lands, grasslands, buildings, roads, structures, excavated lands, deserts, and water areas. Then, we calculated the proportion of each category’s area to the total area in the respective street/town.

We used NPP/VIIRS data from 2019 and 2020. Using the NPP/VIIRS nighttime light data from 2019 as a reference, the nighttime light data from 2020 could be corrected. Specifically, if the light brightness value in 2020 was greater than the light brightness value in 2019, the value remained unchanged. However, if the light brightness value in 2020 was lower, it was assigned the light brightness value of 2019.

The POI data were retrieved from Baidu map, which is one of the most popular commercial map services in China. After data cleaning and filtering, 14 categories of POI data were selected as study objects. We adopted the kernel density estimation (KDE) [56] method to convert discrete individual POIs to continuous and smooth density surfaces for each of the 14 categories. The density surfaces were output as raster layers at a 500 m spatial resolution. The WorldPop dataset was derived from the WorldPop project website (https://www.worldpop.org/). We compared the gridded population map generated by the proposed model with the WorldPop dataset.

## 4. Results

### 4.1. Feature selection results

**Fig 5:**
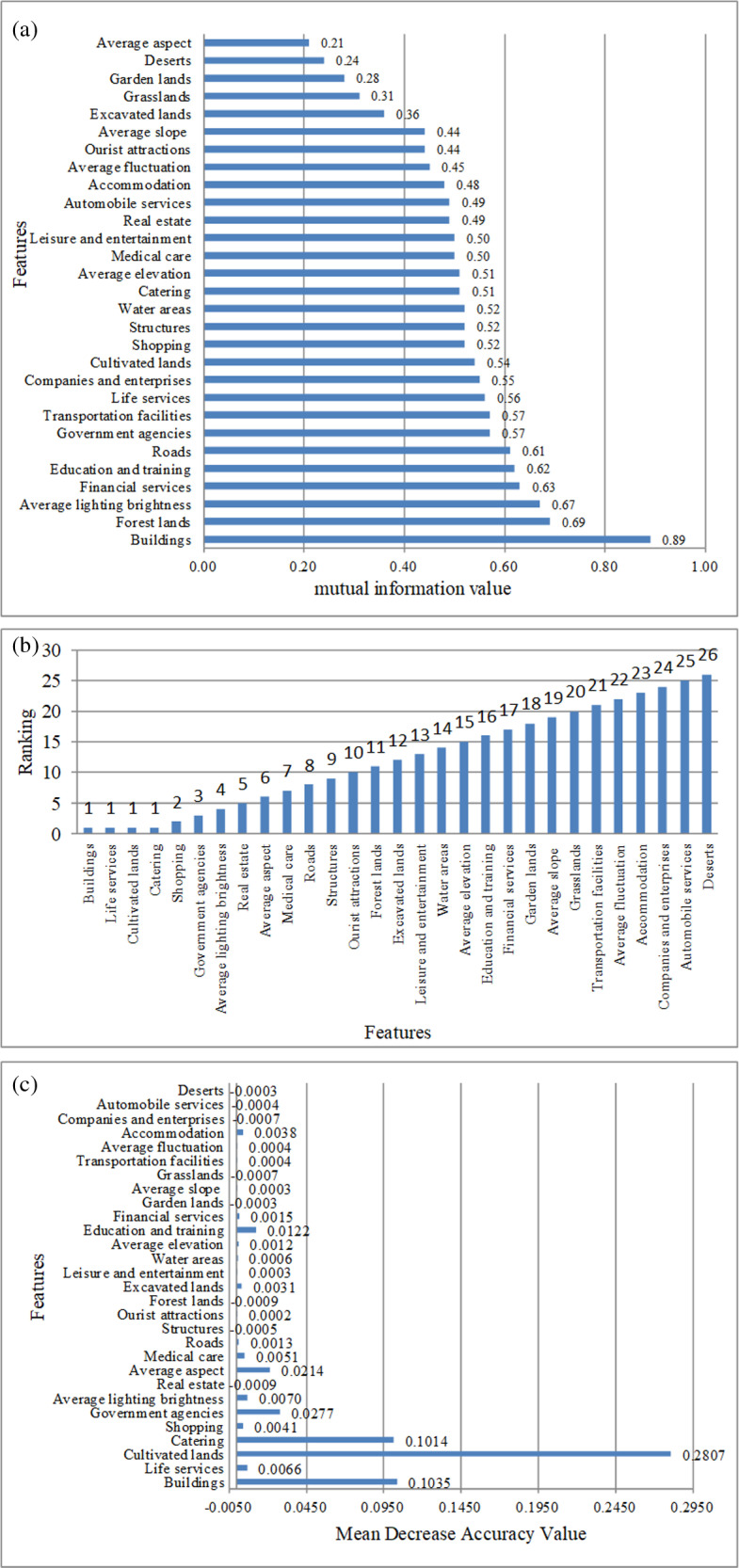
Result of different feature selection methods: (a) feature selection results based on MIC; (b) feature selection results based on RFECV; (c) feature selection results based on MDA.

After data processing, 29 feature factors were derived from the NPP/VIIRS, ASTER GDEM, geographic national census, POI, and administrative district data, including average elevation, average slope, average aspect, average fluctuation, average lighting brightness, cultivated lands, forest lands, garden lands, grasslands, buildings, roads, structures, excavated lands, deserts, water areas, leisure and entertainment, financial services, education and training, life services, government agencies, medical care, automobile services, companies and enterprises, transportation facilities, shopping, accommodation, tourist attractions, catering, and real estate. These features were then subjected to partitioned statistics at the town level administrative units. The MIC, RFE, and MDA methods were used to analyze the relation between the feature factors and population density. Fig 5. shows the feature selection results.

Based on the MIC method, Fig 5(a). is obtained by calculating the importance of each feature to the target variable population density. A larger MIC indicates a stronger correlation between the feature factor and population density, and therefore, a more significant impact on the prediction of population density [38]. Therefore, in this study, the feature factors with a MIC ≥  0.5 were selected for model development, namely buildings, forest lands, average lighting brightness, financial services, education and training, roads, government agencies, transportation facilities, life services, companies and enterprises, cultivated lands, structures, shopping, water areas, catering, leisure and entertainment, and medical care.

Utilizing the RFECV method with a random forest regression model as the feature selection estimator, Fig 5(b). shows the ranking of feature importance obtained through iterative calculations. Among them, catering, life services, buildings, and cultivated lands exhibited the highest feature importance and should thus be considered in the modeling.

Utilizing the MDA method with a random forest regression model as the feature selection estimator, Fig 5(c). shows the average decrease accuracy value for each feature subset. The greater the value of average decrease accuracy, the greater the influence of this feature on the accuracy of the model. Therefore, to ensure that the main feature factors be retained and the minor-influence feature factors be eliminated, the feature factors with a value >  0.004 were selected for model development, including cultivated lands, houses, catering, government agencies, average aspect, education and training, average lighting brightness, life services, medical care, and shopping.

### 4.2. Results of different random forest models

The population density of 435 administrative divisions at the township level was used as the dependent variable. The original features comprised the independent variables. In total, 70% of the sample data were randomly selected from the dataset and used as the training set (305 data points) and the remaining 30% were used as the test set (130 data points). Python language and Scikit-learn open-source library were used as the basis for programming, and a RF regression algorithm was used to construct a population spatialization model and perform model training.

**Fig 6:**
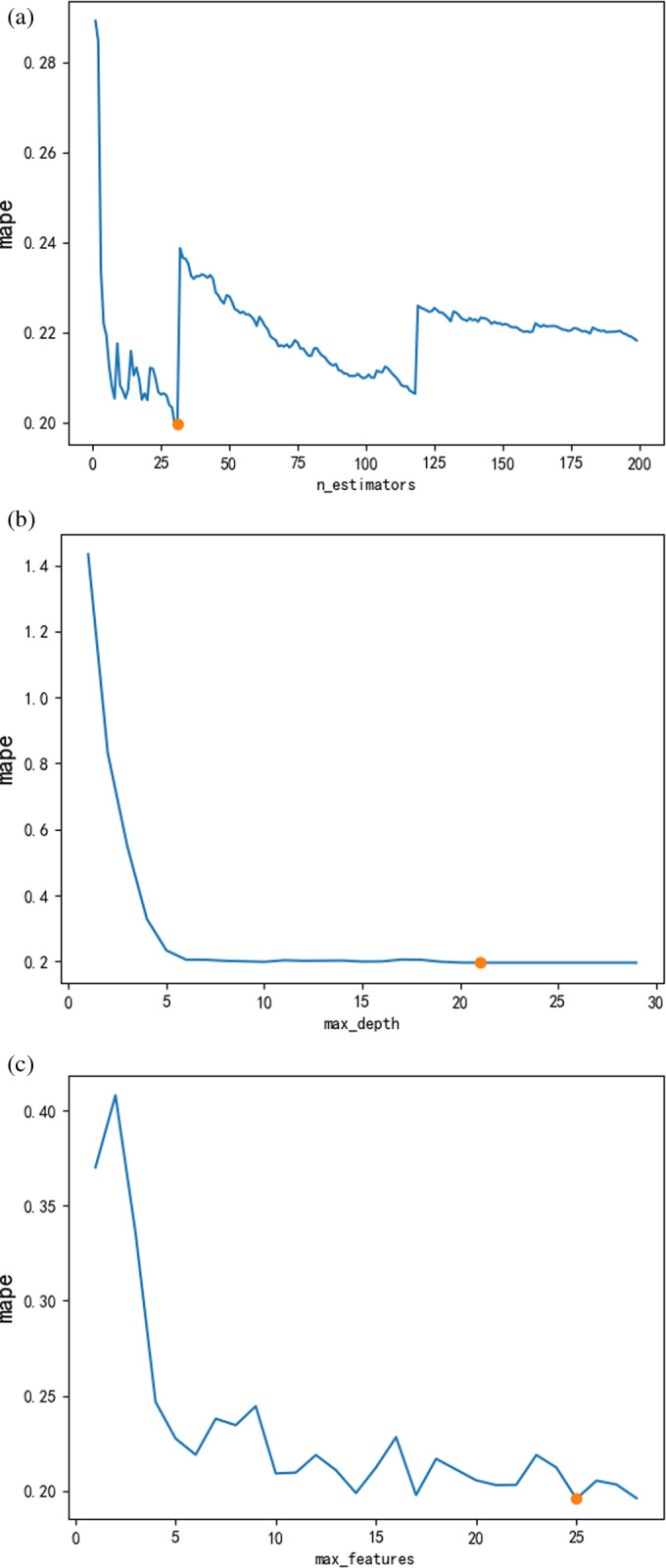
Parameter optimal (a) n_estimators; (b) max_depth; (c) max_features.

When constructing the random forest mode, namely All-RF, based on 29 original features, it is important to determine the optimal hyper-parameters. In this study, we used the grid search method combined with the ten-fold cross-validation technique to train the models and select the best hyper-parameters to ensure that the optimal models were obtained (Fig 6.) [57]. The range and optimal values are shown in Table 2. *n_estimators* is the number of decision trees in the forest, whose optimal value is 31. *max_depth* is the maximum depth of each decision tree, whose optimal value is 22. *max_features* is the number of features randomly chosen at each node, whose optimal value is 25. Similarly, the training and parameter optimization of the random forest model with 18 features selected by MIC, 4 features selected by RFE, and 10 features selected by MDA were completed, i.e., MIC-RF, RFECV-RF and MDA-RF. At the same time, to reduce the influence of random errors in the RF, all the results in this paper are reported as averages across ten experimental runs.

**Table 2:** Paeameter ranges and optimal values.

No	Parameter Value	Value Range	Optimal Value
1	*n_estimators*	[1,200]	31
2	*max_depth*	[1,30]	22
3	*max_features*	[1,29]	25

The test set data were substituted into the above models to predict population density, and the predicted population density values of each street/town under the four models were obtained. To assess the accuracy of the models and the distribution of errors, three evaluation indexes, MAPE, RMSE, and *R*^2^, were calculated for comparison. The results are shown in Table 3. Different models produced different prediction accuracies, with the MAPE range being from 0.174 to 0.203, the RMSE range being from 1416.46 to 1488.67, and the *R*^2^ range being from 0.909 to 0.913. Compared to the All-RF, the other models showed a higher prediction accuracy. In particular, the accuracy improvement of MDA-RF was the largest, at approximately 2.9%. The accuracies of RFECV-RF and MIC-RF were similar; both comprised improvements of around 1.6%. The MDA-RF offered the highest level of correlations between variables, and 91.3% of the population density distribution could be explained using this model, followed by the MIC-RF, the RFE-RF and the All-RF.

**Table 3:** Prediction accuracy of different random forest models.

Model	Number of feature factors	MAPE	RMSE	R^2^
All-RF	29	0.203	1488.67	0.909
MIC-RF	18	0.187	1466.06	0.911
RFECV-RF	4	0.187	1457.21	0.909
MDA-RF	10	0.174	1416.46	0.913

All-RF represents the RF built using all features. MIC-RF, RFECV-RF, and MDA-RF represent the RFs built using the feature selection methods, i.e., MIC, RFECV, and MDA, respectively.

The results indicate that utilizing feature selection methods not only reduces the dimensionality of the model feature dataset, but also improves model accuracy to varying degrees. Of the four models, the MDA-RF had the lowest MAPE of 0.174 and the highest *R*^2^ of 0.913. As demonstrated by the performance variations among ALL-RF, MIC-RF, RFECV-RF, and MDA-RF models, prediction accuracy is highly sensitive to feature factor selection. Redundant or collinear features may inflate complexity without improving accuracy, while oversimplified feature sets risk omitting critical variables. Therefore, considering the accuracy of the model, feature factors selected using the MDA method were used as the optimal feature subset, and subsequent experiments were carried out on this basis.

### 4.3. Improved random forest results

**Fig 7:**
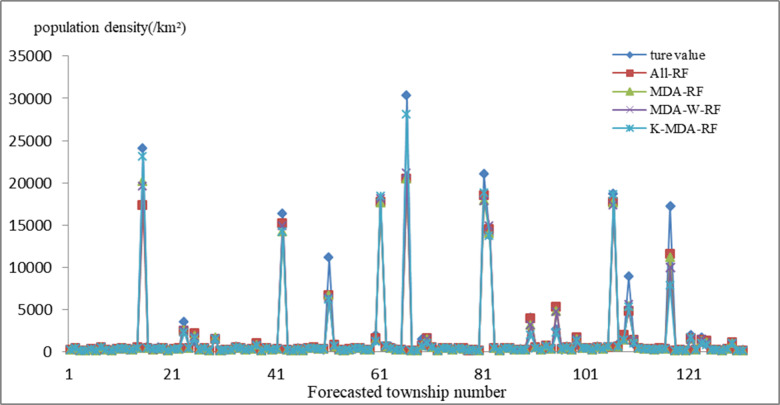
Comparison of predicted and actual values in different models.

The feature factors in the training set were normalized and input into the K-means++ algorithm for clustering. The optimal number of clusters was determined using the elbow method, calculating the sum of squared errors of cluster numbers ranging from 2 to 13 and plotting the corresponding relationship diagram. The results show that when the cluster number k is less than 7, the sum of squared errors drops sharply, whilst with an increase in cluster number k, the sum of squared errors decreases slowly. Therefore, when the training set data were clustered into seven clusters, the best clustering effect was obtained. The bootstrap sampling method was then used to extract the same amount of data from each cluster and fuse the data into a training subset to train the RF. The cross-validation error curve method was used to obtain the optimized model parameters and achieve high model accuracy. The improved RF built using the optimal feature subset was referred to as the K-MDA-RF. Similarly, the weighted random forest built using the optimal feature subset was referred to as the MDA-W-RF. The test set data were substituted into the above models to predict the population density. The prediction accuracy against the actual value was compared in the case of the All-RF, MDA-RF, MDA- W-RF and K-MDA-RF (Fig 7.).

As per Fig 7., the overall prediction results of the K-MDA-RF model are closer to the actual values, and the predicted values of the model are closer to the actual values within the population density range of [0, 5000]. In particular, in streets/towns with extremely high population density, such as NO.14 Guojiaao Street, NO.41 Lianhuachi Street, NO.50 Gongjing Street, NO.60 Daguanlou Street, NO.65 Yuxi Street, NO.80 Nancheng Street, NO.105 Dashanping Street, NO.108 Xiaoxi Street, and NO.116 Loupai Street, the population density prediction values of the K-MDA-RF model were closer to the true values compared to the other two models, reducing the error with the true values to varying degrees.

To further verify the superiority of the K-MDA-RF, its MAPE, RMSE, and *R*^2^ values were calculated, and the results were compared with those of the All-RF、MDA-RF and the MDA- W-RF (see Table 4). Table 4 shows that of the three models, the K-MDA-RF had the lowest MAPE (0.157), and the highest *R*^2^ of 0.939. Compared with the All-RF、MDA-RF and MDA- W-RF, the prediction accuracy of the K-MDA-RF comprised improvements of 4.6%、1.7% and1.9% respectively. Meanwhile, *R*^2^ increased by 3%、1.6% and 1.5%, respectively. Compared with MDA-RF, the prediction accuracy of MDA-G-RF is decreased by 0.2%.

**Table 4:** Evaluation of the improved random forest model.

Model	MAPE	RMSE	R^2^
All-RF	0.203	1488.67	0.909
MDA-RF	0.174	1416.46	0.913
MDA-W-RF	0.176	1443.77	0.914
K-MDA-RF	0.157	1219.51	0.939

In conclusion, compared to the other models evaluated in this study, the population spatialization model of population data constructed based on the K-MDA-RF model yielded better fitting results, smaller prediction bias values, and better relative accuracy of results for population prediction in densely and sparsely populated areas. This indicates that K-means++ clustering algorithm optimized the bootstrap sampling and improved the unevenness of data, thus effectively improving the prediction accuracy of the model.

## 5. Discussions

### 5.1. Population spatialization

**Fig 8:**
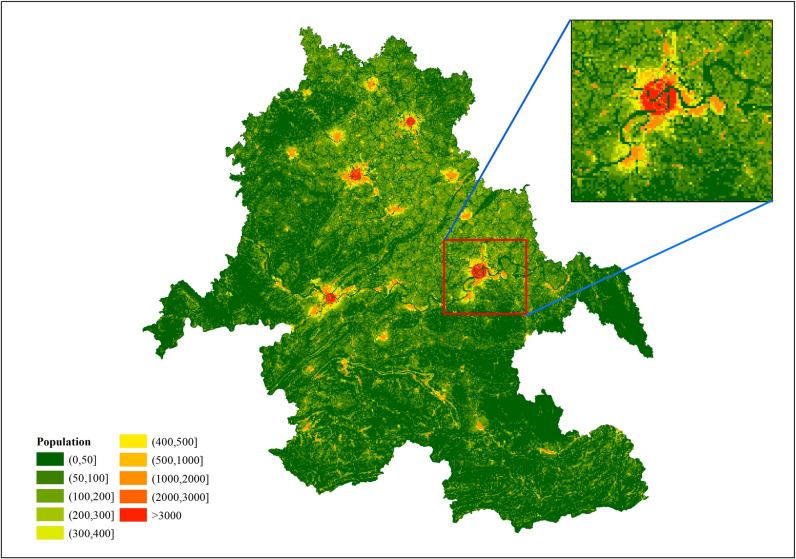
Population distribution in southern Sichuan in 2020 with the 500-m grid. (The base map is based on administrative division data provided by the Sichuan Bureau of Surveying, Mapping and Geoinformation).

The improved random forest model built based on the optimal feature subset was applied to the 500 m grid of the Southern Sichuan Economic Zone to predict the population distribution. The average prediction from the ten experiments was adopted as the final result to generate the spatail population distribution map, as shown in Fig 8. Fig 8 illustrates that the population of the Southern Sichuan Economic Zone was primarily concentrated in the central district area of each city, such as Ziliujing District, Da’an District and Gongjing District of Zigong City, Shizhong District and Dongxing District of Neijiang City, Jiangyang District and Longmatan District of Luzhou City, and Cuiping District of Yibin City. The population concentration decreased gradually as the distance from the central district area increased. The population was densely distributed in the urban center and sparsely distributed in areas away from the urban center, e.g., Zizhong County, Weiyuan County, and Longchang City of Neijiang City; Lu County and Naxi District of Luzhou City; Nanxi District and Jiang’an County of Yibin City; and Fushun County and Rong County of Zigong City. The population distributions in Xuzhou District and Pingshan County of Yibin City and Xuyong County and Gulin County of Luzhou City were sparse and scattered. The population change between the central city and its surrounding towns was more natural and consistent with the actual population distribution. This indicates that the population spatialization model proposed in this article is effective.

### 5.2. Comparison with other datasets

For the analysis of the errors in population spatialization, this study calculated the population of each grid based on the grid-based prediction results and the corresponding grid area. The population was then aggregated at the township/street level as the statistical unit and compared with the WorldPop dataset. The accuracy evaluation results are shown in Table 5. The MRE and RMSE of the WorldPop dataset were 57.24 and 23174.98, respectively, while the MRE (25.00) and RMSE (15776.50) of results for the proposed model were markedly decreased, significantly lower those of the Worldpop dataset. Therefore, it can be concluded that the proposed model better predicted the results than those reported in the WorldPop dataset, confirming the effectiveness and reliability of the proposed model.

**Table 5:** Comparison of model metrics.

Population prediction model	MRE (%)	RMSE
Proposed model	25.00	15776.50
WorldPop dataset	57.24	23174.98

**Fig 9:**
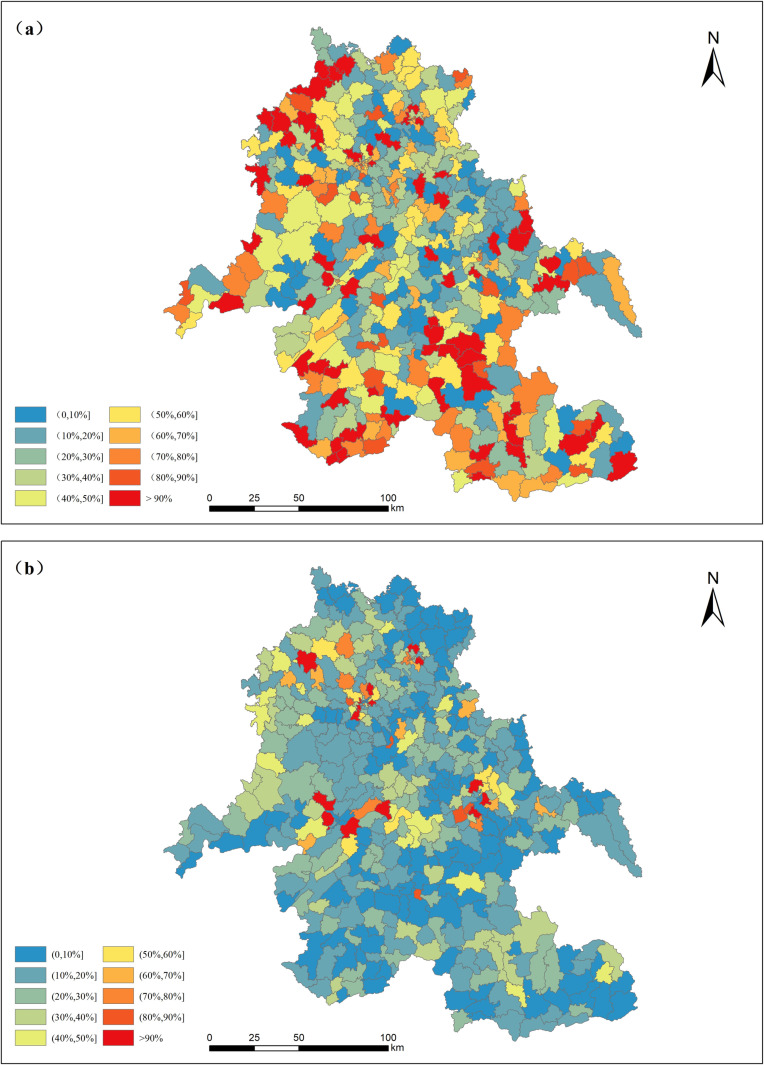
(a) Relative error map of the WorldPop dataset; (b) Relative error map of the proposed model. (The base map is based on the administrative division data provided by the Sichuan Bureau of Surveying, Mapping and Geoinformation. The relative error of population in Fig 9(a). was obtained by summarizing the WorldPop population grid data (https://www.worldpop.org/) and comparing it with the real population of the township).

**Fig 10:**
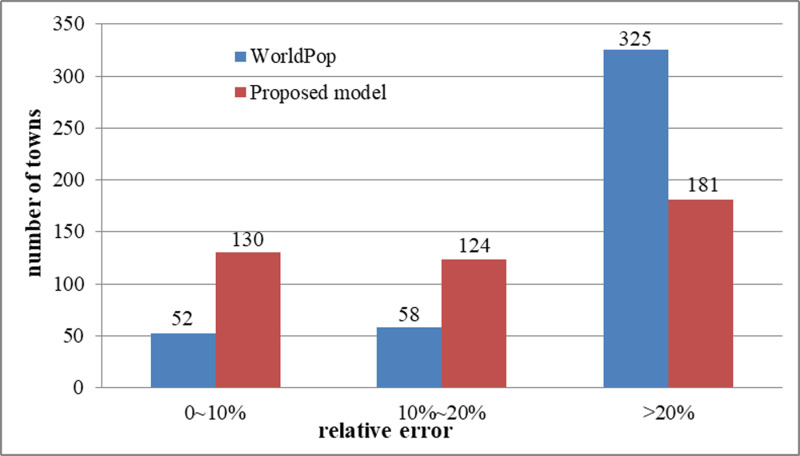
Histogram of error comparison between the proposed model and the Worldpop dataset.

For a more intuitive representation, we compared the relative error between predicted population of the proposed model, the WorldPop dataset and real statistical data. Fig 9(a) and 9(b) show the spatial distribution of the relative errors of the WorldPop dataset and proposed model results. The number of streets or towns falling in each error interval was counted and plotted, as shown in Fig 10.

Fig 9 illustrates that the region with lower relative error of this proposed model is more widely than Worldpop dataset. As depicted in the Fig 9(a)., the relative error in the population distribution of approximately 25% of the townships and streets was less than 20%, while about 19.5% of the townships and streets exhibited relative errors of more than 80%. These areas with large errors are mainly distributed in regions with small populations. However, the relative error in the population distribution of around 60% of townships and streets was less than 20%, while approximately 4.5% of the townships and streets exhibit relative errors of more than 80%.

Fig 10. shows that the errors of the proposed model were concentrated in the range of [0, 20%], covering 254 streets or towns and accounting for 58% of the total number of streets. Notably, the errors for 130 and 124 streets or towns were in the range of [0, 10%] and [10%, 20%], accounting for 30% and 28% of the total number of streets, respectively. This distribution indicates that the predicted population of these streets or towns was very close to the actual values. The prediction errors for 181 streets or towns were > 20%, accounting for 42% of the total number of streets, and those for 14 towns were > 90%. In comparison, the WorldPop dataset had 110 streets or towns in the error range of [0, 20%], accounting for 25% of the total. Notably, the error ranges for 52 and 58 streets or towns were [0, 10%] and [10%, 20%], accounting for 12% and 13% of the total number of streets, respectively. 325 streets or towns in the WorldPop dataset had errors > 20%, accounting for 75% of the total number of streets. Therefore, the number of streets or towns with prediction errors of < 20% determined using the proposed model was considerably higher than in the WorldPop dataset. This indicates that compared to the WorldPop dataset, the dataset generated in this study more accurately represents the spatial distribution of the population.

## 6. Conclusions

Population spatial distribution data is important geographic information; it is of great value in the rational utilization of resources, disaster assessment and post-disaster rescue measures, environmental protection, and urban planning and management. As the available feature factor data related to population distribution gradually increases, the modeling auxiliary factors are no longer singular, and a large amount of feature data is prone to data redundancy. Moreover, the spatial distribution of population data becomes unbalanced. Traditional spatial population data methods yield low accuracy results, making it difficult to use the data to meet actual needs. Therefore, it is necessary to carry out further research on high-precision population spatialization methods.

In this article, we explored and conducted research into population spatialization from two aspects: feature selection and optimized random forest. In terms of feature selection, we utilized RFECV, MMIC, and MDA to select population distribution feature factors. Subsequently, the feature factors corresponding to the model with the highest accuracy were selected as the optimal feature subset and used in model construction as input data.

In terms of optimized random forest, the K-means++ clustering algorithm was utilized to cluster the optimal feature subset. Simultaneously, we selected random forest as the base model. The bootstrap sampling method was used to randomly extract equal numbers of subsample fusions from each type as the training samples of the base learners, and the grid search method combined with the ten-fold cross-validation method, was used to determine the optimal parameters of the model during the training process. The results demonstrated that the optimization of random forest by utilizing the K-means++ clustering algorithm can effectively enhance model accuracy.

This article used the Southern Sichuan Economic Zone as its case study and constructed a spatial population distribution dataset at 500 m resolution based on the best parameter. This distribution aligns closely with the actual population distribution, indicating the effectiveness of the model. When compared to the WorldPop dataset, the RMSE and MRE of the dataset created in this paper were lower, exhibiting higher accuracy and showing the superiority of the modeling approach.

In conclusion, the population spatialization model constructed using the model proposed in this article can effectively predict population distribution. However, the study still has shortcomings and limitations. The accuracy of predictions is inherently tied to the quality, resolution, and completeness of input datasets. For instance, outdated or incomplete population data (e.g., underreported demographics in rural areas) may propagate biases into spatialized outputs. Similarly, reliance on open-source environmental or socio-economic datasets (e.g., satellite imagery, census records) introduces risks of regional inconsistencies or temporal mismatches. The study encountered challenges related to imbalanced datasets. While techniques like classification sampling or weighted mean were applied, their efficacy remains context-dependent and may not fully resolve systemic biases. In addition, the models optimized developed in this study were tailored to specific regions, which may limit their generalizability to other contexts. Variations in environmental, demographic, or socio-economic factors across regions could necessitate recalibration or retraining of the models for optimal performance.

To address these limitations, future research can be carried out from the following aspects. We can integrate multi-temporal, multi-source datasets (e.g., mobile phone data, satellite nightlights) to enhance spatial and temporal coverage. Meanwhile, we can try to synthesize multiple feature selection methods to extract features with higher correlation with population spatial distribution and construct high-precision and high-efficiency refined models. Considering that the degree of imbalance can vary significantly across different geographical regions, researchers should further analyze the proposed method’s performance under different imbalance ratios. In addition, we can investigate transfer learning or meta-learning approaches to improve the generalizability of models across different regions or datasets.
